# *Siglecg* Limits the Size of B1a B Cell Lineage by Down-Regulating NFκB Activation

**DOI:** 10.1371/journal.pone.0000997

**Published:** 2007-10-03

**Authors:** Cheng Ding, Yan Liu, Yin Wang, Bae Keun Park, Cun-Yu Wang, Pan Zheng, Yang Liu

**Affiliations:** 1 Division of Immunotherapy, Section of General Surgery, Department of Surgery, University of Michigan School of Medicine, University of Michigan, Ann Arbor, Michigan, United States of America; 2 Division of Molecular Medicine and Genetics, Department of Internal Medicine, University of Michigan School of Medicine, University of Michigan, Ann Arbor, Michigan, United States of America; 3 Program of Molecular Mechanism of Diseases and Comprehensive Cancer Center, University of Michigan School of Medicine, University of Michigan, Ann Arbor, Michigan, United States of America; 4 Department of Pathology, University of Michigan School of Medicine, University of Michigan, Ann Arbor, Michigan, United States of America; 5 Laboratory of Molecular Signaling and Apoptosis, School of Dentistry, University of California at Los Angeles, Los Angeles, California, United States of America; 6 Integrated Biomedical Graduate Program, The Ohio State University, Columbus, Ohio, United States of America; Washington University, United States of America

## Abstract

**Background:**

B1 B cells are believed to be a unique lineage with a distinct developmental pathway, function and activation requirement. How this lineage is genetically determined remained largely obscure.

**Methods and Principal Findings:**

Using the *Siglecg*-deficient mice with a knockin of green-fluorescent protein encoding sequence, we show here that, although the *Siglecg* gene is broadly expressed at high levels in all stages and/or lineages of B cells tested and at lower levels in other lineages, its deletion selectively expanded the B1a B cell lineages, including the frequency of the B1 cell progenitor in the bone marrow and the number of B1a cells in the peritoneal cavity, by postnatal expansion. The expansion of B1a B cells in the peritoneal correlated with enhanced activation of NFκB and was ablated by an IKK inhibitor.

**Conclusion and Significance:**

Our data revealed a critical role for Siglec G-NFκB pathway in regulating B1a B cell lineage. These data lead to a novel model of B1a lineage development that explains a large array of genetic data in this field.

## Introduction

An important choice a B cell makes is whether to become B1a B cells that produce poly-reactive natural IgM, B1b cells that make adaptive IgM response, or conventional B2 B cells that primarily mediate T-dependent adaptive immunity [1], [2], [3]. While extensive studies have revealed critical roles for the B cell receptor (BCR) recognition, such as its self-reactivity, in determining the lineage choices [4], [5], accumulating evidence indicate that the size of B1 B cells are likely regulated by other factors. For instance, naturally occurring mutations of *Ptpn6* which encodes Shp1 leads to dramatic increase of the B1a B cell compartment [6]. Recent studies using lineage-specific deletion of the *Ptpn6* gene demonstrated a B cell-intrinsic function of SHP1 in limiting the size of the B1a B cells [7]. On the other hand, targeted mutations of *C-Rel* and *NFκB1*, two components of NFκB complex, leads to a significant reduction of the B1 B cells [8]. How these intracellular signaling pathways are used by cell surface molecular interactions to regulate the size of B1 B cell lineage is largely unknown. In particular, targeted mutation of genes encoding several potential membrane proteins that can associate with SHP1, including those of CD72 and CD22, did not substantially impact the development of B1 B cell lineage [9], [10], [11], [12].

Of particular interest are members of the sialic acid-binding, immunoglobulin-like lectin family, or Siglec, many of which have ITIM Motifs for association with SHP1 [13]. Several members of the families, including Siglec 10, the human orthologue of Siglec G, have been demonstrated to be associated with SHP1 [14], [15]. While targeted mutation of Sialoadhesin reduced IgM level, this mutation does not appear to affect the B1 B cell lineage [16]. Siglec G and its human orthologue Siglec 10 appears to have several alternative splicing variants [17], [18] [14], [15]. All variants, however, shared the same cytoplasmic domain with the ITIM motif capable of interacting with SHP-1, which was known to be a negative regulator for B1B cell lineages. Since the function and expression of Siglec G have not been characterized in detail, we produced Siglec G-deficient mice with a knockin of GFP open-reading frame to report its expression. Our results revealed that Siglec G is expressed at high levels at essentially all stages of B cell development and significant but reduced levels in other cell lineages. Importantly, targeted mutation of the Siglec G gene dramatically expanded the B1a B cell lineages postnatally, by increasing NFκB activation. Our data revealed Siglec G is a molecular checkpoint that limits the postnatal expansion of B1a B cells by repressing NFκB activation.

## Results

### GFP-knock-in mice reveal wide-spread expression of *Siglecg* in multiple lineages

Previous studies using flow cytometry have suggested that Siglec 10 may be expressed in subsets of human leukocytes including eosinophils, monocytes and a minor population of natural killer-like cells [18]. Because of the alternative splicing, it is unclear whether these data reflect specific alternative spliced variants. To investigate the cell lineage in which the *Siglecg* locus is active while studying the function of the *Siglecg* in vivo, we replaced essentially all coding exons of the *Siglecg* sequence with a GFP coding sequence, as diagramed in Supplemental Fig. S1A. The genomic DNA clones were isolated from C57BL/6j DNA library for homologous recombination in the ES cells of C57BL/6 origin. The neomycin-resistant ES cell clones were screened for homologous recombination. The clone that was ultimately used for blastocyte injection was shown in Fig. S1B. The chimera mice were continuously bred in B6 background and the offspring shows typical Mendalian distribution of +/+, +/− and −/− genotypes (data not shown). The deletion of the endogenous *Siglecg* genomic sequence is shown in Fig. S1C.

Since the GFP cDNA is fused into exon 2 of the *Siglecg* gene, its expression is controlled by the regulatory elements of the *Siglecg* locus. We therefore used the expression of GFP to report transcription of *Siglecg*. The gene dose effects are measured by comparing the +/− and −/− littermates.

**Figure 1:**
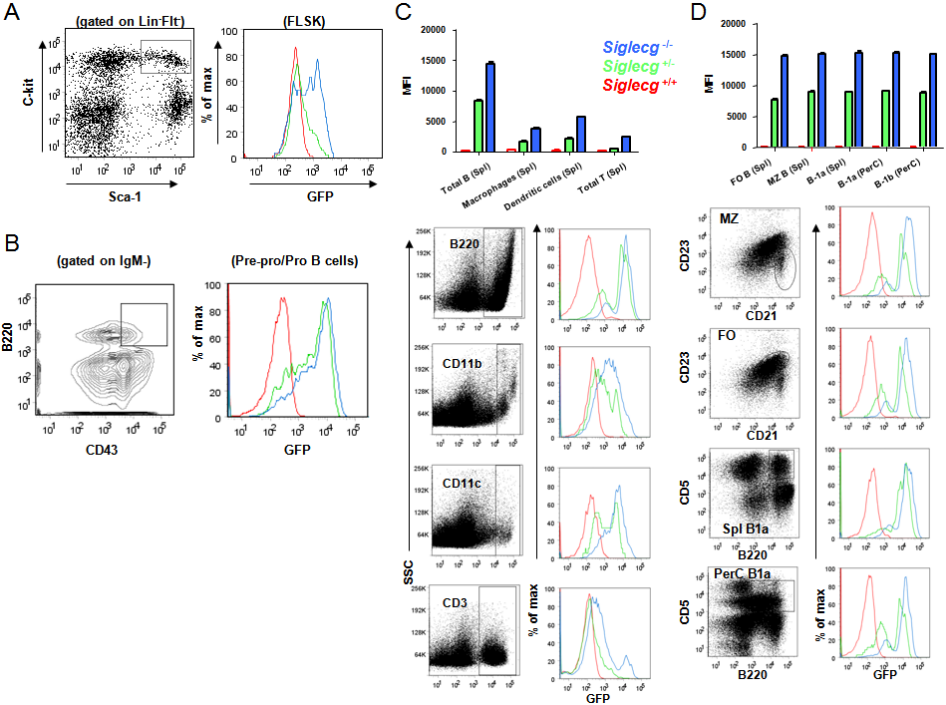
Transcription of the *Siglecg* locus as determined by the expression of GFP which was used to replace the coding sequence of *Siglecg.* Red, WT; green heterozygous; blue, homozygous. A. Low levels of GFP expression among the hematopoeitic cells. The left panel shows the profiles of Lin^−^Flt2^−^Sca-1^+^C-Kit^+^ hematopoeitic stem cells (HSC), while the right panel depicts the GFP intensity of the gated HSC. B. Expression of the *Siglecg* locus in the pre-Pro/Pro B cells. As in A, except that the IgM^−^CD43^+^B220^lo^ cells were depicted. C. Transcription of the *Siglecg* locus among splenic B cells (B220^+^), myeloid cells (CD11b^+^), dendritic cells (CD11c^+^) and T cells (CD3^+^). The top panel shows summary data, while the lower panels show representative FACS profiles. D. The *Siglecg* locus is actively transcribed at all stage/lineages of B cells, as in C. Data shown in this Figure represents 2 mice per group, and has been repeated twice.

The hematopoeitic cells in the bone marrow were identified as FLSK subset, which are Lin^−^Flt2^−^Sca1^+^and c-Kit^+^ [19]. The size of the HSC does not differ among the littermates of the three genotypes (Table 1). Nevertheless, the locus is transcribed in the HSC, albeit at lower levels (Fig. 1A). The Pre-Pro/Pro B cells, as defined by IgM^−^B220^lo^CD43^+^ [20], [21], expressed higher levels of the *Siglecg* transcripts (Fig. 1B). Among the spleen cells, the B cells (B220^+^) expressed the highest levels of *Siglecg* (Fig. 1C). However, significant levels were also detected in DC (CD11c^+^), myeloid cells (CD11b^+^), and to a less extent, T cells (CD3^+^) (Fig. 1C). We further analyzed the GFP levels among the major known B cell subsets. As shown in Fig. 1D, essentially all known subsets expressed high levels of *Siglecg*. Interestingly, heterogeneity of GFP levels was found at all subsets of hematopoeitic cells studied, starting at the HSC.

**Table 1:** Selective expansion of B1a cells in the peritoneal of the *Siglecg-*deficient mice.

	cell population	% of cells	
		*siglecg^−/−^*	*siglecg^+/+^*
Bone marrow	HSC (FLSK) cells	0.085 ± 0.05	0.094 ± 0.04
	Prepro/Pro B (CD43^+^B220^+^IgM^−^)	2.45 ± 0.30	2.81 ± 0.46
	PreB (fr. D) (CD43^−^B220^+^IgMlowIgD^low^)	2.43± 0.39	2.15 ± 0.25
	New B (CD43^−^B220^+^IgM^hi^IgD^low^)	1.36 ± 0.22	1.22 ± 0.22
	Mature B (CD43^−^B220^+^IgM^hi^IgD^hi^)	1.07 ± 0.11	1.30 ± 0.19
	Dendritic cells (CD11c^+^)	1.23 ± 0.09	1.19 ± 0.10
			cell number ( × 10^−6^)
Spleen	Total B (B220^+^)	42.45 ± 8.55	43.99 ± 10.50
	FO B (B220^+^CD23^hi^CD21^hi^)	29.49 ± 3.62	32.16 ± 2.66
	MZ B (B220^+^CD23^low^CD2^hi^)	3.22 ± 0.60	2.55 ± 0.55
	T1 (B220^+^AA4.1^+^CD23^low^IgM^hi^)	6.07 ± 2.88	4.55 ± 1.98
	T2 (B220^+^AA4.1^+^CD23^hi^IgM^hi^)	4.05 ± 1.77	4.89 ± 2.12
	B-1a (B220^+^CD43^+^CD5^+^)	1.97 ± 0.36	2.12 ± 0.39
	Macrophages (CD11b^+^)	4.97 ± 2.02	5.55 ± 1.99
	Dendritic cells (CD11c^+^)	1.32 ± 0.22	1.16 ± 0.33
	T cells (CD3^+^)	23.02 ± 6.99	27.60 ± 8.60
Peritoneal cavity	B-1a (B220^+^CD11b^+^CD5^+^)	2.41 ± 0.51	0.46 ± 0.16
	B-1b (B220^+^CD11b^+^CD5^−^)	1.07 ± 0.25	1.25 ± 0.33
	T cells (CD3^+^)	0.97 ± 0.11	1.11 ± 0.29

The cell subsets were phenotyped as described [27]. For BM HSC cells, N = 3. For spleen and peritoneal cavity T cells, N = 6. For all other cells, N = 12.

Taken together, our data demonstrated that the *Siglecg* locus is transcribed in essentially all the major cell types in the hematopoeitic cell types analyzed, although B cells, as a group, seem to have the highest levels.

### Targeted mutation of *Siglecg* leads to preferential expansion of B1a B cells in the peritoneal cavity

**Figure 2:**
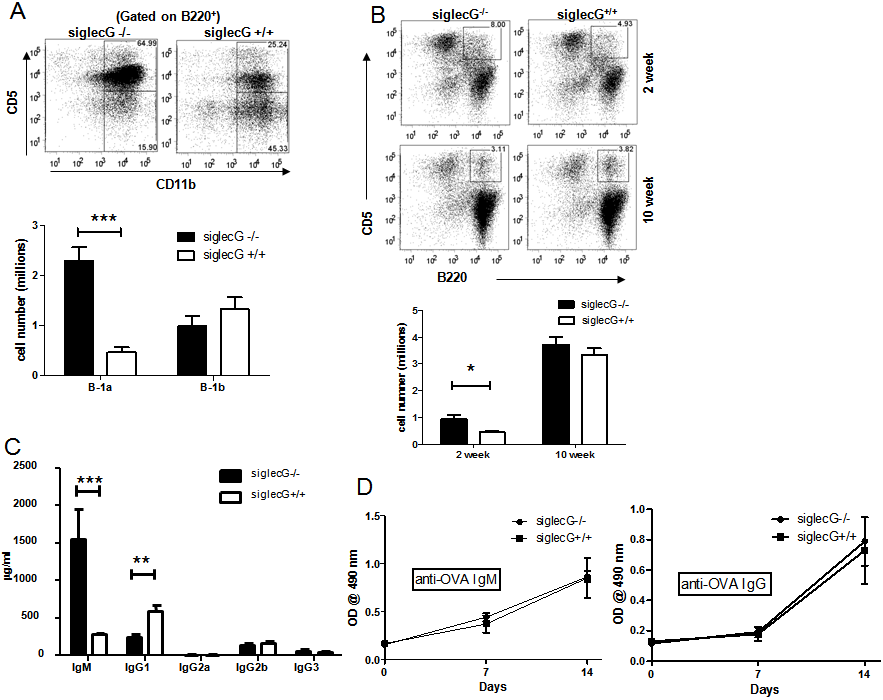
Selective expansion of peritoneal cavity B1a cells in mice with targeted mutation of the *Siglecg* gene. A. Expansion of B1a, but not B1b subsets. The top panel shows the profile of B cells among peritoneal lavages of 10 weeks old mutant and age-matched WT controls, while the lower panel shows summary data involving 10 mice per group. This experiment has been repeated 5 times involving a total of 10–12 mice per group. C. Serum concentration of different isotypes of Ig, note substantial increase of IgM and decrease of IgG1 in the mutant mice. Data shown in the lower panels are Means+SEM, with 20 mice per group. D. Adaptive B cell response in WT and the *Siglecg-*deficient mice. The WT and *Siglecg-*deficient mice were immunized with 200 µg of OVA in complete Freunds' adjuvants (CFA) on day 0, boosted with the same amounts of OVA on day 7. Sera were collected on day 1, 7 and 14 for measurement of anti-OVA antibodies by ELISA using the OVA-coated plate. Data shown are means+SEM of optical density, n = 6. Sera were used at 1∶500 dilutions for the ELISA.

Given the wide-spread expression of *Siglecg*, we determined whether targeted mutation of the gene affects the development of different compartments of the hematopoeitic cells by flow cytometry. As shown in table 1, the compositions of the major hematopoeitic subsets, including HSC, T cells, myeloid cells, dendritic cells and different stages of B cells, were comparable between the WT and the mutant mice. Surprisingly, we have observed a 5-fold increase in the size of B1a cells in the peritoneal, although the number of B1b subset was unchanged (Fig. 2A). The number of B1a cells in the spleen, however, did not differ in adult mice (Fig. 2B lower panel and table 1), although a two fold expansion of spleen B1 B cells can be observed at two weeks (Fig. 2B, upper panel).

Corresponding to the expansion of the B1a cells, the levels of serum IgM increased by 5-10 fold (Fig. 2C). With exception of IgG1, which is reduced by approximately 2-fold, the levels of other Ig isotypes was unaffected by *Siglecg* deletion. To determine whether deletion of *Siglecg* affects adaptive B cell responses, we immunized the WT and *Siglecg-*deficient mice with OVA and determined the antibody levels at one and two weeks after immunization. As shown in Fig. 2D, the antibody response was essentially unaffected by the *Siglecg* deletion.

### Postnatal expansion of B1a progenitors in the *Siglecg^−/−^* mice

It is generally believed that the B1a progenitors give rise to mature B1a cells during fetal development and that self-renewal of the B1a cells after birth explains the long-life persistence of the B1a lineage [3]. More recently, a low number of B1a progenitor cells were identified in mature bone marrow [22]. In order to explain the drastic difference in the number of B1a cells in the peritoneal cavity, we carried out BrdU labeling to determine the proliferation of the B1a cells. As shown in Fig. S2, at two weeks, when a significant difference was observed in the % of B1a B cells found in the spleen, the rate of BrdU incorporation was the same in the two groups. Conversely, at 10 weeks, when no difference in the B1a subsets was observed in the spleen, there appeared to be a higher rate of BrdU incorporation in the *siglecg-*deficient mice. Furthermore, in the peritoneal cavity, where a dramatic difference of the number of B1a cells were found, the rate of BrdU incorporation was essentially identical. Thus, while our data did not rule out the possibility that *Siglecg* may regulate B1a proliferation in specific locations, such function does not account for the differential accumulation of B1a cells in the *Siglecg-*deficient mice. Likewise, the % of Annexin V^+^ cells was also comparable (Fig. S3). Thus, neither increased self-renewal nor decreased apoptosis of mature B1a cells explain the expansion of the B1a compartment.

**Figure 3:**
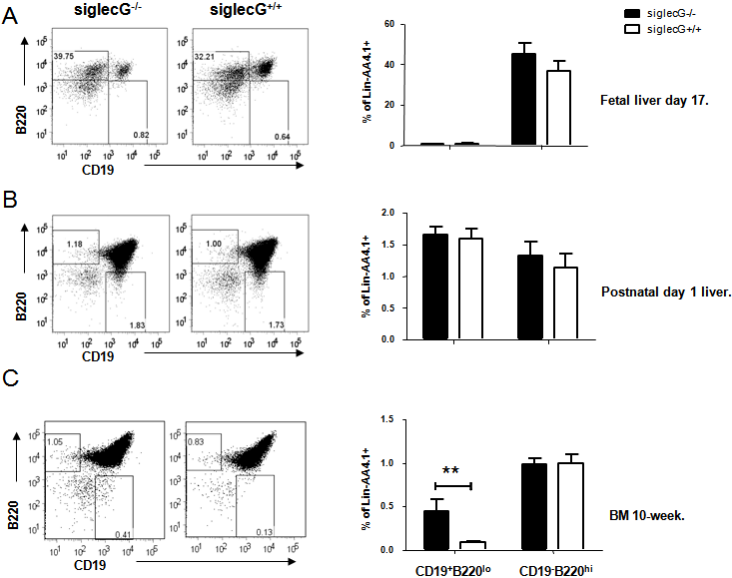
Postnatal expansion of B1a but not B2 progenitor cells. A. Progenitor cells from fetal liver. B. Progenitor cells from new born liver cells. C. Percentage of B1 (CD19^+^B220^lo^) and B2 (CD19^−^B220^hi^). Data shown in A involve 2 WT and 2 mutant mice per group. Data shown in B involve 3 WT and 4 mutant mice per group. Similar levels were found in 12 heterozygous littermates. C. As in A, except 10 week old bone marrow were analyzed. N = 7.

To explore the possibility that increased number of B1a cells may be due to the expansion of the B1 progenitors, we compared the number of the progenitors in the fetal liver and adult bone marrow. The Lin^−^AA4.1^+^ liver and bone marrow mononuclear cells can be further divided into 4 subsets by their expression of B220 and CD19. Among them, CD19^+^ B220^lo^ subsets are considered the B1a progenitors, while the CD19^−^B220^hi^ subsets are regarded as the B2 progenitors [22]. As shown in Fig. 3A, the number of B1a progenitors in the E16.5–E17 fetal livers was unaffected by *Siglecg* mutation. Likewise, the number of B1a progenitor in the newborn livers was also similar between WT and mutant mice (Fig. 3B). In contrast, the number of B1 progenitors was expanded by 4-fold in the bone marrow of adult *Siglecg-*deficient mice, in comparison to what was found in the WT littermates (Fig. 3C). In either compartment, the B2 progenitors were not affected by *siglecg* mutation (Fig. 3A–C).

**Figure 4:**
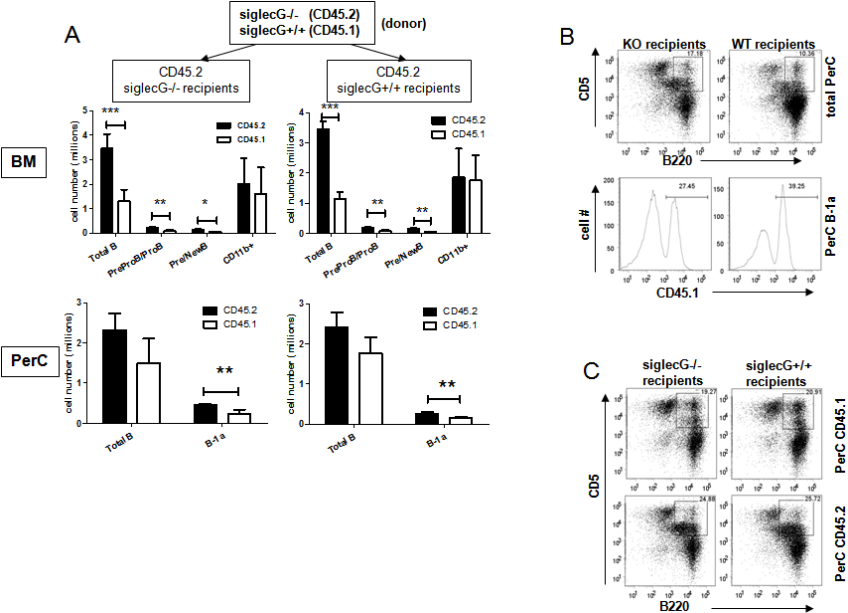
Siglecg-deficient bone marrow cells have competitive advantages in reconstituting bone marrow B cell lineage and peritoneal B1a subsets in lethally irradiated recipients. The WT (CD45.1) and *Siglecg* ^−/−^ (CD45.2) bone marrow cells were mixed at a 1∶1 ratio and used to reconstitute the lethally irradiated mice, 13 weeks later, the mice were sacrificed for analysis. The bone marrow and peritoneal lavages were analyzed by flow cytometry for reconstitution by WT and mutant bone marrow cells. A. Summary data showing the contribution of WT (open bars) and mutant bone marrow cells (filled bars). The cell subsets in bone marrow include total B cells (B220^+^), Pre-pro/Pro B (CD43^+^B220^lo^sIgM^−^) and pre/new B (CD43^−^B220^+^IgM^low-high^IgD^low^). The lower panel summarizes the total B cells and B1a B cells in the peritoneal lavages. Data shown are mean+SEM, N = 5. B. Representative profiles depicting the relative contribution of WT (CD45.1) and *Siglecg*-deficient (CD45.2) cells to the B1a compartment. C. Representative profiles depicting distinct profile of WT and Siglecg-deficient B1a cells.

To determine whether the adult bone marrow from the *siglecg*-deficient mice have increased tendency to reconstitute the B1a compartment, we mixed equal numbers of total bone marrow cells from *Siglecg^+/+^* (CD45.1) and the *Siglecg^−/−^* (CD45.2) mice, and adoptively transferred them into lethally irradiated WT or *Siglecg^−/−^* host. Unlike the mice with B cell-specific deletion of *Ptpn6* [7], no expansion of B1a cells was found in the bone marrow was found in the *Siglecg^−/−^* mice (Fig. S4), therefore no effort was made to delete the B1a cells from bone marrow. Thirteen weeks after bone marrow transplantation, the bone marrow, peritoneal cavity and spleen were harvested and analyzed by flow cytometry. As shown in Fig. 4A, regardless of the recipient genotypes, the *Siglecg^−/−^* bone marrow were more efficient in reconstituting the B cell compartment, including Pre-ProB/Pro and Pre/new B cells, and the total number of B cells. In contrast, bone marrows from the two genotypes were comparably efficient in reconstituting the myeloid compartment.

Importantly, in the peritoneal cavity, the number of *Siglecg^−/−^* B1a cells was 2–3 fold more abundant than that from the WT bone marrow, while the number total B cells were not significantly different (Fig. 4A lower panel and Fig. 4B). In addition to the difference in the total number, an interesting difference in the cell surface markers was also observed. Thus, the majority of the B1a cells derived from WT bone marrow expressed high CD5, essentially all of the B1a cells from the mutant bone marrow exhibited low levels of CD5. The increased number of progenitor cells in the adult bone marrow, but not in fetal liver, and over-presentation of mutant B1a cells in the peritoneal cavity strongly suggests that the expansion of the B1a cells in the peritoneal cavity of the *Siglecg^−/−^* mice was due to postnatal expansion of the B1a cells, perhaps due to increased frequency of B1a progenitor cells. Interestingly, unlike the peritoneal cavity, the number of B1a cells in the spleen is mainly derived from the WT donor (Fig. S5). Such phenotype is reminiscent of a recently reported phenotype associated with SHP-1 mutation in the B cell compartment [7].

### Activation of NFκB is essential for the expansion of the B1a compartment in the peritoneal cavity of the *Siglecg^−/−^* mice

**Figure 5:**
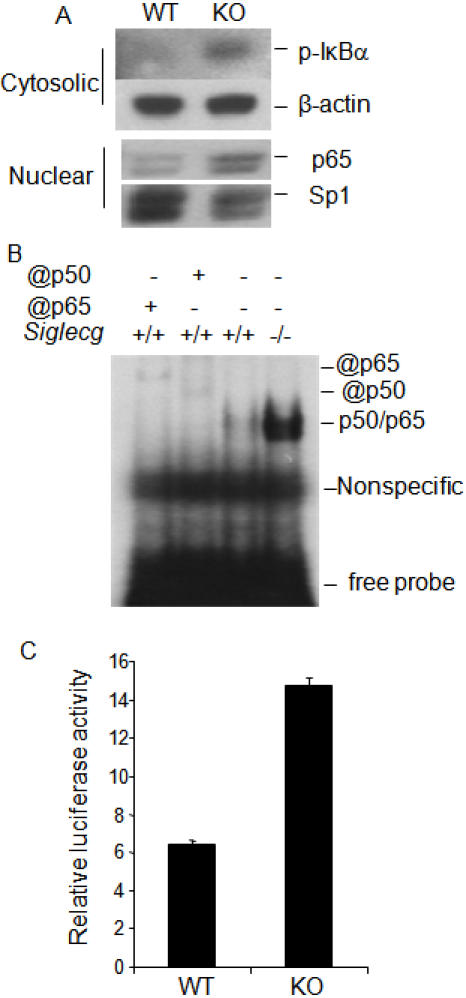
*Siglec^−/−^* peritoneal cavity lavage cells have increased NFκB activity. A. Western blot analysis of cytosolic total and phorsphorylated IkBa (upper panels) and nuclear P65, using the amounts of SP1 as loading control (lower panels). B. EMSA analysis of nuclear NFκB activity. Nuclear lysates from WT (+/+) or knockout peritoneal lavages were prepared and tested for retardation of ^32^P NFkB probe. The specificity of the gel retardation was confirmed by super-shift with anti-P50 or P65 antibodies. C. Luciferase activity of peritoneal cells from WT and knockout mice. Data shown in this figure have been repeated twice.

We compared ex vivo WT and *Siglecg-*deficient peritoneal cavity cells for activation of a number of signal transduction pathways, including phosphorylation of Akt, Stat 3, Stat 5, P38, Erk and JNK. Our extensive analyses fail to reveal a significant difference between the WT and mutant cells (Data not shown). Since mutation of *Ptpn6*, which associate with Siglecg orthologue Siglec 10, enhanced NFκB activation and expanded B1a compartment [6], and since mice with targeted mutation of components in NFκB pathway show selective reduction of B1a B cell compartment [8], we focused on the potential impact of *Siglecg* deletion on NFκB activation. As shown in Fig. 5A, ex vivo peritoneal lavage from *Siglecg*-deficient mice show greatly increased IκBα phosphorylation in the cytosol and nuclear accumulation of P65, as demonstrated by Western blot (Fig. 5A). This corresponds to greater binding to NFκB probe as revealed by mobility shift assays (Fig. 5B). Furthermore, the increased NFκB function was revealed by luciferase assay when the peritoneal cells from WT and *Siglecg*-deficient mice were compared (Fig. 5C).

**Figure 6:**
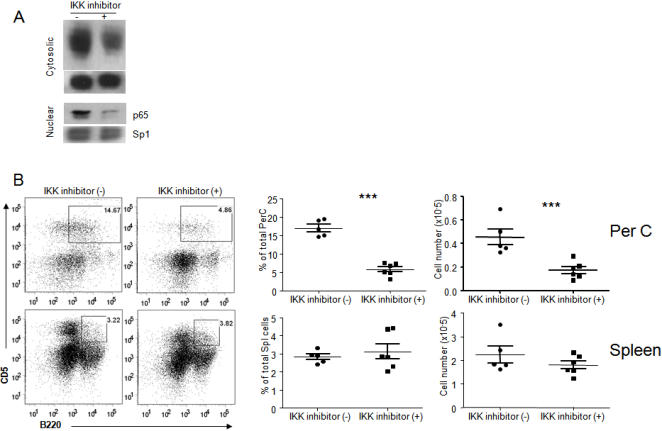
IKK inhibitor treatment decreased B-1a cells in peritoneal cavity but not spleen. (A). Efficacy of the treatment in reducing IKK activity among total spleen cells as revealed by IκBα phosphorylation in the cytosol (upper panel) P65 accumulation in the nuclei (lower panel) of spleen cells from treated or control mice. B. The impact of inhibiting the IKK on peritoneal B1a subsets. Data shown are means+SEM involving 5–6 mice per group.

The increased IκBα phosphorylation indicated enhanced IKK activity in the B1a cells from the *Siglecg^−/−^* peritoneal cavity. To determine whether the activation of NFκB is essential for the expansion of the B1a compartment, we treated the 5-days old *Siglecg*-deficient mice with IκB kinase complex (IKK) inhibitor VI, which we have demonstrated to work efficiently in the mice [23]. As shown in Fig. 6A, consecutive treatment resulted in greatly decreased IκBα phosphorylation and nuclear P65 accumulation. Importantly, the percentage and number of B1a cells in the peritoneal cavity was reduced by 3–4 folds in the treated group. In contrast, despite effective inhibition of IKK, the number of B1a cells in the spleen was unaffected by the inhibitor (Fig. 6B). These results reveal that activation of the NFκB pathway is essential for selective expansion of the B1a cells in the peritoneal cavity of the *Siglecg^−/−^* mice.

## Discussion

A long-standing observation in immunology is the preferential accumulation of B1a B cell in the peritoneal cavity. Given the importance of the B1a B cells in innate immunity and autoimmune diseases [1], [2], [3], the mechanisms that determine the size of B1a B cell subsets in the locality are of great interest. Using mice with targeted mutation of the *Siglecg* gene, we demonstrate that *Siglecg* controls B1 B cell expansion in the peritoneal cavity by repressing NFκB activation.

### *Siglecg* controls a checkpoint for peritoneal B1a B cell expansion

We have observed mice with targeted mutation of *Siglecg* that had a 5–10 fold increase in serum IgM, while those of most other Ig isotypes were roughly normal. In search for a cellular basis for the over-production of IgM, we uncovered the dramatic expansion of the B1 B cells in the peritoneal cavity. Of the two subsets of B1 B cells in the cavity, the expansion is limited to the CD11b^+^ B1a subsets. Moreover, while the expansion of the B1a B cells in the peritoneal cavity were observed throughout the study, the expansion of the B1 B cells in the spleen is only transient, observable at 2 weeks but not 10 week.

Since no reagent is currently available to study the protein expression for mouse *Siglecg*, we used a GFP knockin transgenic line to determine the expression of *Siglecg*. Our data indicated that while cells among B cell lineage had the highest levels of *Siglecg* expression, essentially all major subsets of hematopoeitic cells examined, including T cells, DC, and monocytes show active transcription of the locus. Despite the wide-spread expression of the gene, the impact of *Siglecg* deletion appear to be limited to the B1a B cells in the peritoneal cavity, although a smaller expansion of the B1a B cells in the spleen can be observed at 2 weeks of age.

While our manuscript was in preparation, Hoffmann et al. [24] reported a *Siglecg-*deficient mouse prepared with ES cells from the BALB/c. While they observed expansion of B1a cells in the spleen and peritoneal cavity, the B1a expansion observed in our study is more selective. The difference in background genes between the two strains of mice may account for the difference.

### Postnatal expansion of B1a progenitors and expansion of B1 B cells in the *Siglecg*-deficient mice

While the B1a B cells are generally believed to have been produced from progenitors in fetal liver [3], recent studies identified B1a progenitors in adult bone marrow [22] [25]. Therefore, the expansion of the B1a B cell compartment can theoretically be attributed to either increased fetal production or postnatal production. Our analysis of adult progenitor cells in the bone marrow and fetal livers indicated a substantial increase in the B1a B progenitors in the bone marrow, but not in the fetal or new born liver. Consistent with the increased B1a B progenitor cells, the *Siglecg-*deficient bone marrow are more efficient in constituting the B1a B cells. These data, together with the fact that neither increased proliferation nor decreased apoptosis explains the expansion of B1a B cells in the peritoneal cavity, supports the notion that postnatal expansion of the B1a compartment is responsible for the expansion of B1a cells in the *Siglecg^−/−^*mice. Therefore, our data demonstrate a novel function of *Siglecg* in regulating postnatal expansion of the B1a compartment. It remains to be determined as to whether the expansion is intrinsic to genetic defects in the precursor cells, or due to *Siglec*g defects in other cell types.

It is worth pointing out that although the number of progenitors for B2 cells were not increased, the *siglecg*-deficient bone marrow was more efficient in reconstituting B cells than the WT bone marrow when they were mixed with WT bone marrow cells. These data suggest that in addition to the number of B1 B progenitors, *Siglecg* exerts a general repressive effect on the differentiation of the B cell compartment. Such requirement, however, seems to be reflected in competition with progenitor cells from the WT host, but not with other cell lineage, as the relative number of B cells are the same when the *Siglecg-*deficient mice were analyzed.

### *Siglecg* is a negative regulator of NF*κ*B activity in peritoneal B cells: similarity with *Shp1*

Our comparison between WT and *Siglecg-*deficient ex vivo peritoneal lavage revealed that activation of a number of signaling pathways, including AKT, STAT, Erk, P38 and JNK was not affected by the mutation (data not shown). In contrast, a profound activation of NF*κ*B was observed in the peritoneal lavages from the *Siglecg-*deficient mice in comparison to that from WT mice, as indicated by increased phosphorylation of IκB, nuclear localization of P65, increasing mobility shift of the NFκB probe and increased promoter activity when the NFκB reporter constructs were used to transfect the peritoneal lavage cells. Since the activation correlates with the IκB phosporlation, it is likely that the conical NFκB pathway is being activated. Moreover, since the IKK is responsible for IκB phosphorylation, we used the IKK inhibitor VI to block NFκB activation in order to study the significance of this activation in B1a cell expansion in the peritoneal cavity. Our data clearly demonstrated that activation of NFκB is responsible for the expansion of the B1a cells in the peritoneal cavity. Recently, Hoffmann et al. reported that mutant B cells display increased Ca^2+^ signaling in responses to several stimuli in vitro [24]. Since it is unclear if these stimuli are responsible for the B1a expansion in vivo, the significance of the enhanced Ca^2+^ response remains to be demonstrated.

Interestingly, despite the significant effect of the IKK inhibitor for spleen cells, no increase in the spleen B1a B cells were observed. These data clearly indicated that expansion of B1a B cells in the peritoneal cavity uses a mechanism that is not employed to maintain the number of spleen B1a B cells. The selective expansion of B1a B cells from the mutant peritoneal cavity suggests that the expansion requires both unique checkpoint in the specific cell lineage and specific stimuli in the peritoneal cavity. Moreover, we have observed no effect of IIKK inhibitor on BrdU incorporation in B1a cells (data not shown). However, since our data also indicated that the expansion cannot be demonstrated by BrdU incorporation (Fig. S2), the cellular basis for the increase remains to be determined.

Recently, Rajewsky and colleagues demonstrated that targeted mutation of *Shp1* in the B cell lineage causes expansion of B1a B cells in the peritoneal cavity [7]. Remarkable similarity can be found between the germline mutation of the *Siglecg* and B-cell specific deletion of *Ptpn6.* Firstly, in terms of lineage size, the impact of mutation is limited within B1a B cells in the peritoneal cavity. Secondly, the expansion of B1a subsets is due to the postnatal expansion of B1a cells. Thirdly, in bone marrow chimera mice, the mutant bone marrow shows a remarkable advantage in reconstituting the peritoneal B1a B cells, while displaying a disadvantage in constituting spleen B1a B cell compartment. These similarities in functional defects raise the possibility that the gene has a similar function within B cell compartment. Since Siglc10, the Siglec G orthologue in human, has been shown to be associated with Shp1[14], [15], the simplest hypothesis is that Shp1 works down-stream of Siglec G in limiting the controlled expansion of B1a subset. This hypothesis has the potential to unify observations made from mice with genetic defects of *Siglecg*, *Ptpn6*, and *NFκB1* and *C-rel*. Since mutation of *Siglecg* increases the titer of anti-DNA antibodies [24] and since deletion of Shp1 in B cells causes autoimmune diseases [7], the balance of the Siglecg signaling will likely be important in the proper tuning of the B1a function in innate immunity against infection vs. autoimmune side effect. Given recent advances in pharmaceutical targeting of the NFκB pathway [23], our study suggests a new approach in selective tuning of innate immunity and autoimmunity.

## Materials and Methods

### Generation of *Siglecg^−/−^GFP^+/+^* mice using C57BL/6 ES cells

The production of the *Siglecg^−/−^GFP^+/+^* mice is carried out by the Ingenious Research Laboratory, Inc. (Long Island, New York) under a research contract. Detail information is provided by Fig. S1. Briefly, a ∼9.8 kb region used to construct the targeting vector was first sub cloned from a positively identified BAC clone using a homologous recombination-based technique. The region was designed such that the short homology arm (SA) extends 1.8 kb 3′ to exon 11. The long homology arm (LA) ends in exon 2 just before the ATG and is ∼8.8 kb long. The GFP/Neo cassette (Neo is flanked by both loxP and FRT sites) is inserted before the ATG of Exon 2 and replaces ∼5 kb of the gene sequence including exons 3–11.

Ten micrograms of the targeting vector was linearized by AscI and then transfected by electroporation of iTL C57/Bl6 embryonic stem cells. After selection in G418, surviving clones were expanded for PCR analysis to identify recombinant ES clones. Primers, A1, 2 and 3 were designed downstream (3′) to the short homology arm (SA) outside the region used to generate the targeting construct. PCR reactions using A1, 2 or 3 with the N1 primer at the 5′ end of the Neo cassette amplify 2.0, 2.0 and 2.1kb fragments respectively. The control PCR reaction was done using AT1 and AT2, which is at the 5′ end of the SA inside the region used to create the targeting construct. This amplifies a band of 1.7 kb. As shown in Fig. S1B, Individual clone was screened with A1/N1, A2/N1 and A3/N1 primers. ES clone #361 was identified as a recombinant clone and injected into BALB/c blastcytes. The chimera mice were bred to B6 mice to obtain F1 mice, which were intercrossed to generate *Siglecg^−/−^GFP^+/+^* mice, and their WT and heterozygous littermates.

### Isotype specific ELISA

Serum immunoglobulin titers were determined by enzyme-linked immunosorbent assay (ELISA) using BD Falcon plates (BD biosciences) coated with goat anti-mouse immunoglobulin (2 µg/ml; Southern Biotechnology, Birmingham, AL) diluted in sodium bicarbonate buffer (pH 8.2). Standard curves of each Ig isotype were generated with purified mouse IgM, IgG1, IgG2a, IgG2b and IgG3 (Southern Biotechnology). Plates were blocked with 1% non-fat milk in PBS for 1 h at room temperature, and sera were diluted in blocking buffer (1% non-fat milk in PBS) (IgM and IgG1, 1∶10,000; IgG2a, IgG2b and IgG3,1∶5,000) and allowed to bind to the plate for 2 hours at room temperature. Horseradish isotype-specific antibodies (Southern Biotechnology) were then added after washing with 0.05% Tween 20 in PBS and then substrate Sigma Fast OPD (Sigma Aldrich, St. Louis, MO). Optical densities at 490 nm were measured with a fluorescence plate reader (SpectroMax 190, Molecular Devices, Toronto, Canada).

### Flow cytometry

Single-cell suspensions were prepared from tibial bone marrow, spleen and peritoneal cavity from mice euthanized with CO_2_. Cells were suspended in RPMI 1640. Prior to staining, red cells in spleens were lysed (BD Pharm Lyse, BD biosciences). Cells were then suspended in PBS with 2% FBS and 1% sodium azide for staining. Cells were surface stained for 30 minutes at 4^o^C and intracellular staining of BrdU is done following manufacturer (BD Pharmingen) provided protocol. Cells were then suspended in staining buffer and transferred for flow cytometry (BD LSR II, BD biosciences). Analysis of the flow cytometry data was done with Flowjo 7.2. Antibodies used were either purchased from BD Pharmingen (La Jolla, CA): (B220, APC-Cy7 streptavidin, c-kit, Sca-1, CD21, CD5, CD19, and Lin markers, CD3, Gr-1, CD11b, Ter119) or purchased from EBioscience (La Jolla, CA), CD23 and AA4.1.

### Western blot

Cells were lysed by regular lysis buffer containing 1% Triton-X100, 1∶100 diluted, protein and phosphatase cocktails ( both from Sigma). Nuclear and cytoplasmic proteins were isolated by a nuclear and cytoplasmic protein extraction kit (Pierce Biotechnology, Rockford, IL). Antibodies for western-blot are phospho-antibodies of p-AKT, p-GSK3β, p-p38, p-p42/44, p-JNK, p-IκB, p-p65, p-Stat3, and p-Stat-5, all from Cell Signaling, and corresponding control antibodies of GSK3α/β, IκBα, from Cell Signaling (Davers, MA), and those specific for p-65, and Sp1 from Santa Cruz Biotechnology (Santa Cruz, CA).

### Electrophoresis mobility shift assay

Nuclear extracts were prepared as described previously [26]. 10-µg aliquots of cell nuclear extracts were pre-incubated with 1 µg of poly(dI-dc) in binding buffer (10 mM Tris [pH 7.7], 50 mM NaCl, 20% glycerol, 1 mM dithiothreitol [DTT], 0.5 mM EDTA) for 10 min at room temperature. Approximately 1.5×10^4^ cpm of ^32^P-labeled DNA probes were then added and the reaction proceeded for 30 min. The specificity of the binding was further confirmed by anti-P65 or anti-P50 antibodies (Santa Cruz). The sequence for the probe was 5′-AGTTGAGGGGACTTTCCCAGGC-3′. The sequence for the probe was 5′-AGTTGAGGGGACTTTCCCAGGC-3′. The complexes were resolved on a 5% polyacrylamide gel in Tris-glycine buffer consisting of 25 mM Tris, 190 mM glycine and 1 mM EDTA at room temperature. The gel was dried at 80°C for 60 min and exposed to an X-ray film.

### NFκB activity reporter assay

Peritoneal cells extracted from WT and the Siglecg−/− mice were cultured in RPMI medium containing 10% FBS for overnight. Next day, the cells were transfected with NF-κB reporter plasmid of NF-κB-TA-luc (Clontech, San Diego, CA) by using Lipofectamine 2000 in 10% FBS Opti-MEM medium for 16 hours, and recovered in fresh complete RPMI medium for additional 12 to 16 hours. Plasmid Renilla luciferase (PRLSV40) was used as internal control for monitoring transfection efficiency.

### Bone marrow chimera

Bone marrow cells from WT and the *Siglecg−/−* mice were mixed at a 1∶1 ratio (2.5×10^6^ each) and were injected into lethally irradiated (1100 RAD) WT or Siglecg^−/−^ mice. The bone marrow, spleen, lymph node and peritoneal lavages were harvested at 13 weeks after irradiation and analyzed by flow cytometry.

### In vivo treatment with IKK inhibitor

5-day old siglecG^−/−^ mice were treated with 0.01 mg of IKK inhibitor VI (Calbiochem, San Diego, CA) intra-peritoneally twice daily for 8 days and spleen and peritoneal cavity cells were harvested for analysis.

### Statistics

The statistical significances of observed differences were analyzed by student t-tests. *, 0.05>P>0.01; **, 0.01>P>0.001; ***, P<0.001.

## Supporting Information

Figure S1 Generation of Siglecg−/− mice that expressed green fluorescence protein (GFP) under the control of Siglecg regulatory sequence. A. Diagram of construct (top), siglecg genomic structure (middle) and the recombinant knock-out/in allele (bottom). Top, diagram of the knock-out/in construct. LA, long arm that ended before the Siglecg coding sequence in exon 2, SA, short arm consisting of 1.8 kb intron 2 sequence. GFP coding sequence is linked to exon 2 with its own stop codon and polyadenylations sites. Neo sequence is transcribed from the opposite direction. Middle, genomic structure, shaded area indicates coding sequence from exon 2 to 12. Bottom, the structure of the knock-in allele. The primers used are marked. B. Verification of homologous recombination by PCR. Data shown were for clone 361, which was used to generate the knockout/in mice. Integration of the construction is confirmed by AT1/N1 primer pair, while integration is confirmed by A1/N1, A2/N1 and A3/N1 pairs. The AT1/AT2 pair was the positive control for PCR. The primer sequences are: Primers used: A1: agctaagcacatgtgatggcta, A2: aggtgaataagtataggcccggc, A3: tgtgtgacctcaaggttgctc, AT1: tcagagtctcacttaccactcc, AT2: tattcagaggagtctgtggc, N1: tgcgaggccagaggccacttgtgtagc. C. Genotyping of the F2 mice using primers that identify WT, heterozygous and homozygous knockout/in mice. Primers used: siglecg-F: tcccagacttgcatgagaatc, silgecg-R: atgttctctctggaccagagg, GFP-F: atgtgatcgcgcttctcgtt, GFP-R: gagcgcaccatcttcttcaa. Product size: sigecg: 800bp, GFP: 280bp.
(2.72 MB TIF)

Figure S2 Preferential proliferation of mature B1a cells does not explain the expansion of B1a compartment in the Siglecg-deficient mice. A. Proliferation of splenic B1 B cells of 2- (top) and 10 (bottom)-weeks old mice. B. Proliferation of peritoneal B1a B cells from 10 week old mice. Two or 10 weeks-old Siglecg−/− and Siglecg+/+ mice were injected (ip) with 1 mg of BrdU 27 and 3 hours before sacrifice. Spleen and peritoneal B-1 a proliferation is analyzed by BrdU incorporation. The gates used were shown in the left. Profiles of BrdU incorporation is shown in the middle, while the summary data are shown in the right. Data shown are representative of 3 independent experiments.
(3.46 MB TIF)

Figure S3 Siglecg does not control apoptosis of the B1B cells. Data shown are histograms depicting annexin V staining of ex vivo splenic (A) or peritoneal B1a B cells of 10-week old mice. The gates applied are the same as Fig. S2.
(3.11 MB TIF)

Figure S4 No increase of B1a B cells in bone marrow in the Siglecg−/− mice.
(0.92 MB TIF)

Figure S5 Siglecg-deficient bone marrow cells have competitive disadvantages in reconstituting spleen B1 subsets in lethally irradiated recipients. Spleen cells used were from chimera mice described in Fig. 4 legends.
(2.61 MB TIF)
