# Supplementary figures and images for: Siglecg Limits the Size of B1a B Cell Lineage by Down-Regulating NFκB Activation
Source: PLoS One. 2007 Oct 3;2(10):e997. doi: 10.1371/journal.pone.0000997 (PMC1994585; doi:10.1371/journal.pone.0000997)

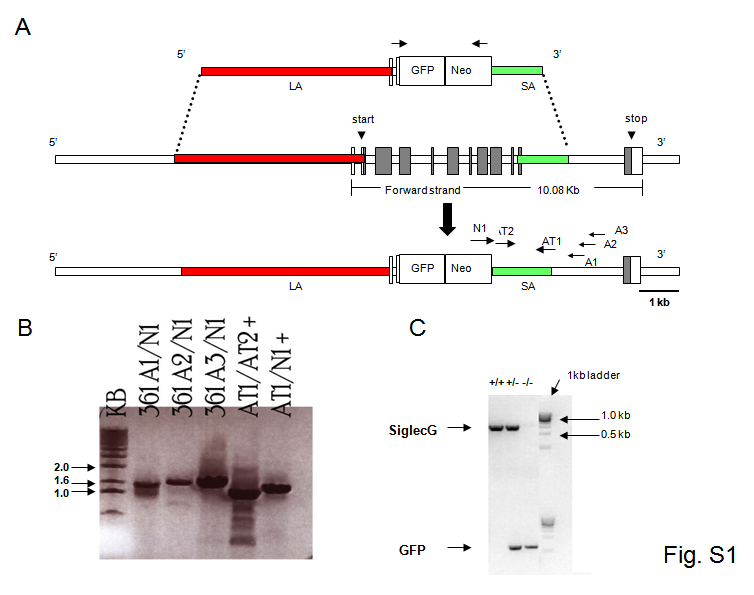

Supplement: Figure S1 — Generation of Siglecg−/− mice that expressed green fluorescence protein (GFP) under the control of Siglecg regulatory sequence. A. Diagram of construct (top), siglecg genomic structure (middle) and the recombinant knock-out/in allele (bottom). Top, diagram of the knock-out/in construct. LA, long arm that ended before the Siglecg coding sequence in exon 2, SA, short arm consisting of 1.8 kb intron 2 sequence. GFP coding sequence is linked to exon 2 with its own stop codon and polyadenylations sites. Neo sequence is transcribed from the opposite direction. Middle, genomic structure, shaded area indicates coding sequence from exon 2 to 12. Bottom, the structure of the knock-in allele. The primers used are marked. B. Verification of homologous recombination by PCR. Data shown were for clone 361, which was used to generate the knockout/in mice. Integration of the construction is confirmed by AT1/N1 primer pair, while integration is confirmed by A1/N1, A2/N1 and A3/N1 pairs. The AT1/AT2 pair was the positive control for PCR. The primer sequences are: Primers used: A1: agctaagcacatgtgatggcta, A2: aggtgaataagtataggcccggc, A3: tgtgtgacctcaaggttgctc, AT1: tcagagtctcacttaccactcc, AT2: tattcagaggagtctgtggc, N1: tgcgaggccagaggccacttgtgtagc. C. Genotyping of the F2 mice using primers that identify WT, heterozygous and homozygous knockout/in mice. Primers used: siglecg-F: tcccagacttgcatgagaatc, silgecg-R: atgttctctctggaccagagg, GFP-F: atgtgatcgcgcttctcgtt, GFP-R: gagcgcaccatcttcttcaa. Product size: sigecg: 800bp, GFP: 280bp. (2.72 MB TIF) [file pone.0000997.s001.tif]

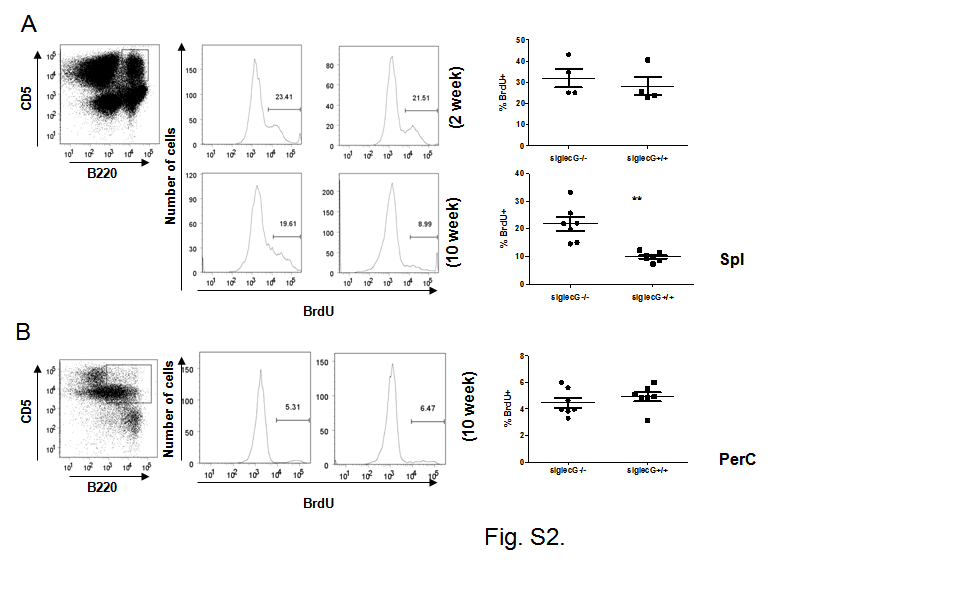

Supplement: Figure S2 — Preferential proliferation of mature B1a cells does not explain the expansion of B1a compartment in the Siglecg-deficient mice. A. Proliferation of splenic B1 B cells of 2- (top) and 10 (bottom)-weeks old mice. B. Proliferation of peritoneal B1a B cells from 10 week old mice. Two or 10 weeks-old Siglecg−/− and Siglecg+/+ mice were injected (ip) with 1 mg of BrdU 27 and 3 hours before sacrifice. Spleen and peritoneal B-1 a proliferation is analyzed by BrdU incorporation. The gates used were shown in the left. Profiles of BrdU incorporation is shown in the middle, while the summary data are shown in the right. Data shown are representative of 3 independent experiments. (3.46 MB TIF) [file pone.0000997.s002.tif]

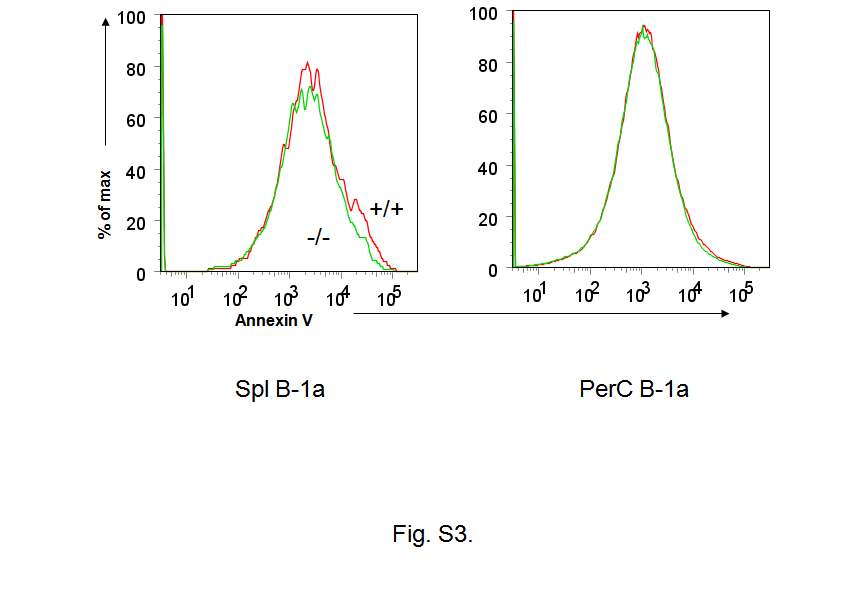

Supplement: Figure S3 — Siglecg does not control apoptosis of the B1B cells. Data shown are histograms depicting annexin V staining of ex vivo splenic (A) or peritoneal B1a B cells of 10-week old mice. The gates applied are the same as Fig. S2. (3.11 MB TIF) [file pone.0000997.s003.tif]

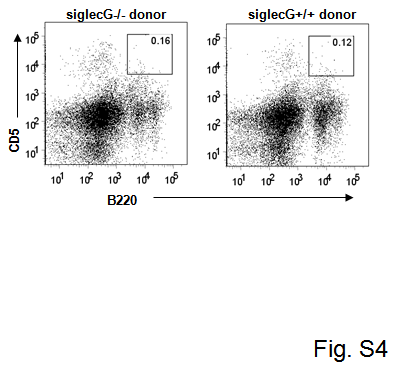

Supplement: Figure S4 — No increase of B1a B cells in bone marrow in the Siglecg−/− mice. (0.92 MB TIF) [file pone.0000997.s004.tif]

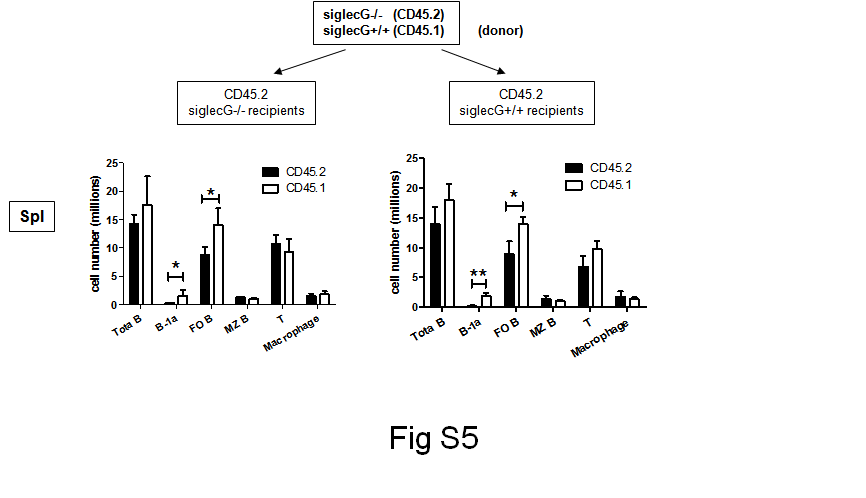

Supplement: Figure S5 — Siglecg-deficient bone marrow cells have competitive disadvantages in reconstituting spleen B1 subsets in lethally irradiated recipients. Spleen cells used were from chimera mice described in Fig. 4 legends. (2.61 MB TIF) [file pone.0000997.s005.tif]
